# A *Listeria monocytogenes* ST2 clone lacking chitinase ChiB from an outbreak of non-invasive gastroenteritis

**DOI:** 10.1080/22221751.2018.1558960

**Published:** 2019-01-16

**Authors:** Sven Halbedel, Rita Prager, Sangeeta Banerji, Sylvia Kleta, Eva Trost, Gopala Nishanth, Georg Alles, Christina Hölzel, Friederike Schlesiger, Ariane Pietzka, Dirk Schlüter, Antje Flieger

**Affiliations:** a FG11 Division of Enteropathogenic Bacteria and Legionella, Robert Koch Institute, Wernigerode, Germany; b German Federal Institute for Risk AssessmentBerlin, Germany; c Institute of Medical Microbiology and Hospital Hygiene, Otto-von-Guericke University Magdeburg, Magdeburg, Germany; d Institute of Medical Microbiology and Hospital Epidemiology, Hannover Medical School, Hannover, Germany; e Paderborn District, Health Office, Paderborn, Germany; f Faculty of Agricultural and Nutritional Sciences, CAU Kiel, Kiel, Germany; g Milk Hygiene, Faculty of Veterinary Medicine, LMU Munich, Oberschleißheim, Germany; h Chemical and Veterinary Analytical Institute Ostwestfalen-Lippe (CVUA-OWL), Detmold, Germany; i German-Austrian Binational Consiliary Laboratory for Listeria, Austrian Agency for Health and Food Safety (AGES), Vienna, Austria; j Organ-specific Immune Regulation, Helmholtz Centre for Infection Research, Braunschweig, Germany

**Keywords:** Molecular surveillance, core genome MLST, chitin, listeriosis, invasion

## Abstract

An outbreak with a remarkable *Listeria monocytogenes* clone causing 163 cases of non-invasive listeriosis occurred in Germany in 2015. Core genome multi locus sequence typing grouped non-invasive outbreak isolates and isolates obtained from related food samples into a single cluster, but clearly separated genetically close isolates obtained from invasive listeriosis cases. A comparative genomic approach identified a premature stop codon in the *chiB* gene, encoding one of the two *L. monocytogenes* chitinases, which clustered with disease outcome. Correction of this premature stop codon in one representative gastroenteritis outbreak isolate restored chitinase production, but effects in infection experiments were not found. While the exact role of chitinases in virulence of *L. monocytogenes* is still not fully understood, our results now clearly show that ChiB-derived activity is not required to establish *L. monocytogenes* gastroenteritis in humans. This limits a possible role of ChiB in human listeriosis to later steps of the infection.

## Introduction

*Listeria monocytogenes* is a Gram-positive intracellularly replicating bacterium widespread in the environment. *L. monocytogenes* infects humans by ingestion of contaminated food, predominately through milk, fish and meat products but also vegetables. It represents an important food-borne human pathogen that can cause severe invasive disease with a high fatality rate of about 20–30% [1]. The number of human listeriosis cases has been increasing during the last years in Germany as well as in Europe [2]. *L. monocytogenes* may cause large outbreaks and sources of infection are difficult to identify due to the lengthy incubation time [3]. Therefore, the bacterium has a considerable economic impact and requires great public health attention and action measures such as pathogen surveillance.

The infectious dose of listeriosis was estimated to be 10^7^–10^9^ CFU for the normal and 10^5^–10^7^ for the high risk population [4]. Highly susceptible for infection are immunocompromised individuals, pregnant women, foetuses, neonates, and the elderly (>60 years) [5,6]. Severe *L. monocytogenes* infections occur when the bacterium manages transition from the gut lumen into the blood stream, from where it disseminates to the liver, the brain and the placenta in pregnant women. Such invasive situations lead to the following clinical symptoms: foetal infections, encephalitis, meningitis, and septicaemia [7] and are characterized by incubation times of 1–67 days [3]. In immunocompetent individuals a non-invasive form of listeriosis characterized by febrile gastroenteritis, flu-like symptoms and shorter incubation time varying from 6 to 240 h may occur [3,8–12], but the pathogenic mechanisms leading to gastroenteritis are not well understood [9].

A set of virulence factors has been described for *L. monocytogenes*, of which the genes encoded on *Listeria* pathogenicity island-1 (LIPI-1) are the best characterized. These include the regulator PrfA, the two phospholipases PlcA and PlcB, listeriolysin O (LLO), the metalloproteinase Mpl and the actin-polymerization inducing ActA. LLO is the main driver of bacterial egress from primary and secondary vacuoles, and a member of the cholesterol-dependent cytolysin (CDC) toxin family [13]. Both phospholipases and Mpl support listerial escape from vacuoles [14] and the surface protein ActA facilitates the polymerization of cytoplasmic actin [15,16] and thereby drives cell-to-cell spread [17]. Two internalins InlA and InlB, located elsewhere on the chromosome, are required for host cell invasion [18,19]. Furthermore, auxiliary factors, such as the chitinases ChiA and ChiB, are required for full virulence in mice [20]. Both enzymes act together to degrade chitin, a structural component of the cell wall of fungi and the exoskeleton of arthropods [21]. Chitinase expression is induced during growth in the soil [22], suggesting that these enzymes serve nutrient acquisition during life in the environment. However, secretion of ChiA is also important for intracellular survival of *L. monocytogenes* as ChiA secretion is linked to reduced expression of the inducible nitric oxide synthase (iNOS) [23], a major factor of the host immune response.

*L. monocytcogenes* isolates from approximately two thirds of all known notification cases are collected by the German-Austrian binational consiliary laboratory for *Listeria* located at the German Robert Koch Institute and the Austrian Agency for Health and Food Safety Ltd (AGES) each year. In parallel, isolates from food sources are collected and typed by the national reference laboratory for *L. monocytogenes* at the German Federal Institute for Risk Assessment. These labs have recently introduced whole genome sequencing (WGS) and core genome multi locus sequence typing (cgMLST) for cluster detection and this has greatly improved recognition of listeriosis outbreaks [24–26]. In spring 2015, a cluster of *L. monocytogenes* isolates from a local gastroenteritis outbreak in Germany and associated food sources was detected. As disease mechanisms in gastroenteritis due to *L. monocytogenes* infection are not well understood [9], this study describes the characterization of the *L. monocytogenes* clone, which has caused this outbreak using WGS-based subtyping, comparative genomics, genetic approaches and virulence studies.

## Results

### *L. monocytogenes* isolates from an outbreak of non-invasive listeriosis among preschool children

An outbreak of non-invasive listeriosis occurred among 163 patients, among which were 152 children (1–9 years, median 5 years) and 11 adults (17–56 years, median 38 years), that all suffered from gastrointestinal symptoms after consumption of rice pudding from a single local catering service in March 2015 in the Paderborn district in North-East North-Rhine Westfalia/Germany. Molecular typing of 44 *L. monocytogenes* isolates from affected patients revealed molecular serogroup IVb and identical PFGE profiles after macrorestriction with AscI or ApaI (Figure S1, Table 1). This PFGE profile had not been detected before and was given the internal AscI/ApaI profile number 17b/25var, corresponding to The European Surveillance System (TESSy) code 329/495. The 44 isolates had been isolated from stool samples of 37 young children (2–7 years of age) and one adult (35 years), all suffering from gastroenteritis. Isolate pairs were obtained from two of the children with gastrointestinal symptoms by consecutive sampling (isolates 15-01121 and 15-01424 as well as isolates 15-01129 and 15-01500). An additional 17b/25var clone (15-01429) was identified by the consiliary laboratory at the same time, but this clone was isolated from a blood sample of a 72-year-old patient with invasive listeriosis, who was living in southern North-Rhine Westfalia. Epidemiological investigations (data not shown) identified several food types including rice pudding from a local caterer, contaminated with high loads (up to 1.5 × 10^7^ CFU/g) of *L. monocytogenes* serogroup IVb with the 17b/25var PFGE profile, as the most probable source of infection (Table 1).

**Table 1.** *L. monocytogenes* isolates genome sequenced in this study.

Internal code	Source of isolation	Patient age	Disease	Molecular serogroup^a^	PFGE AscI/ApaI^b^	cgMLST CT^c^	ENA accession no.
*Clinical isolates*							
07-00066	Cerebrospinal fluid	64	Deceased	IVb	17b/25	2361	ERS2102984
08-00118/1	Blood	74	Unknown	IVb	17a/10	2403	ERS2102985
09-02927	Unknown	69	Unknown	IVb	17b/25	1112	ERS2102988
13-00439	Blood	Unknown	Unknown	IVb	17b/188	2401	ERS2473683
13-02266	Cerebrospinal fluid	41	Meningitis	IVb	17b/25	1113	ERS2103020
14-05759	Blood	63	Brain abscess	IVb	17bvar/240	2400	ERS2103055
14-06478	Blood	77	Sepsis	IVb	17a/25a	2402	ERS2103060
15-01121	Stool	2	Gastroenteritis	IVb	17b/25var	1114	ERS2103082
15-01128	Stool	2	Gastroenteritis	IVb	17b/25var	1114	ERS2103084
15-01129	Stool	5	Gastroenteritis	IVb	17b/25var	1114	ERS2103085
15-01130	Stool	5	Gastroenteritis	IVb	17b/25var	1114	ERS2103086
15-01131	Stool	5	Gastroenteritis	IVb	17b/25var	1114	ERS2103087
15-01132	Stool	5	Gastroenteritis	IVb	17b/25var	1114	ERS2103088
15-01331	Stool	5	Gastroenteritis	IVb	17b/25var	1114	ERS2103093
15-01393	Stool	4	Gastroenteritis	IVb	17b/25var	1114	ERS2103097
15-01424	Stool	2	Gastroenteritis	IVb	17b/25var	1114	ERS2103098
15-01429	Blood	72	Sepsis, fever	IVb	17b/25var	1634	ERS2103099
15-01500	Stool	5	Gastroenteritis	IVb	17b/25var	1114	ERS2103102
15-01527	Stool	5	Gastroenteritis	IVb	17b/25var	1114	ERS2103103
15-01528	Stool	3	Gastroenteritis	IVb	17b/25var	1114	ERS2103104
15-01591	Stool	5	Gastroenteritis	IVb	17b/25var	1114	ERS2103107
*Food isolates*							
Internal code	Source of isolation^d^						
15-01745	Rice pudding			IVb	17b/25var	1114	ERS2473683
15-01746	Rice pudding			IVb	17b/25var	1114	ERS2473684
15-01747	Rice pudding			IVb	17b/25var	1114	ERS2473685
15-01748	Rice pudding			IVb	17b/25var	1114	ERS2473686
15-01750	Chopped chicken and red cabbage			IVb	17b/25var	1114	ERS2473687
15-01751	Rice			IVb	17b/25var	1114	ERS2473688
15-01752	Rice			IVb	17b/25var	1114	ERS2473689
15-01753	Rice			IVb	17b/25var	1114	ERS2473690
15-01754	Rice			IVb	17b/25var	1114	ERS2473691
15-01755	Sausage in curry sauce			IVb	17b/25var	1114	ERS2473692

^a^Molecular serogroups were determined by multiplex PCR [52,53].

^b^PFGE was performed using the PulseNet protocol (https://www.cdc.gov/pulsenet/pdf/listeria-pfge-protocol-508c.pdf).

^c^cgMLST was performed according to Ruppitsch et al*.* [27].

^d^These food isolates were found in different foods that were all produced by the same local catering service.

### Subtyping of outbreak isolates by genome sequencing and core genome MLST

**Figure 1.**
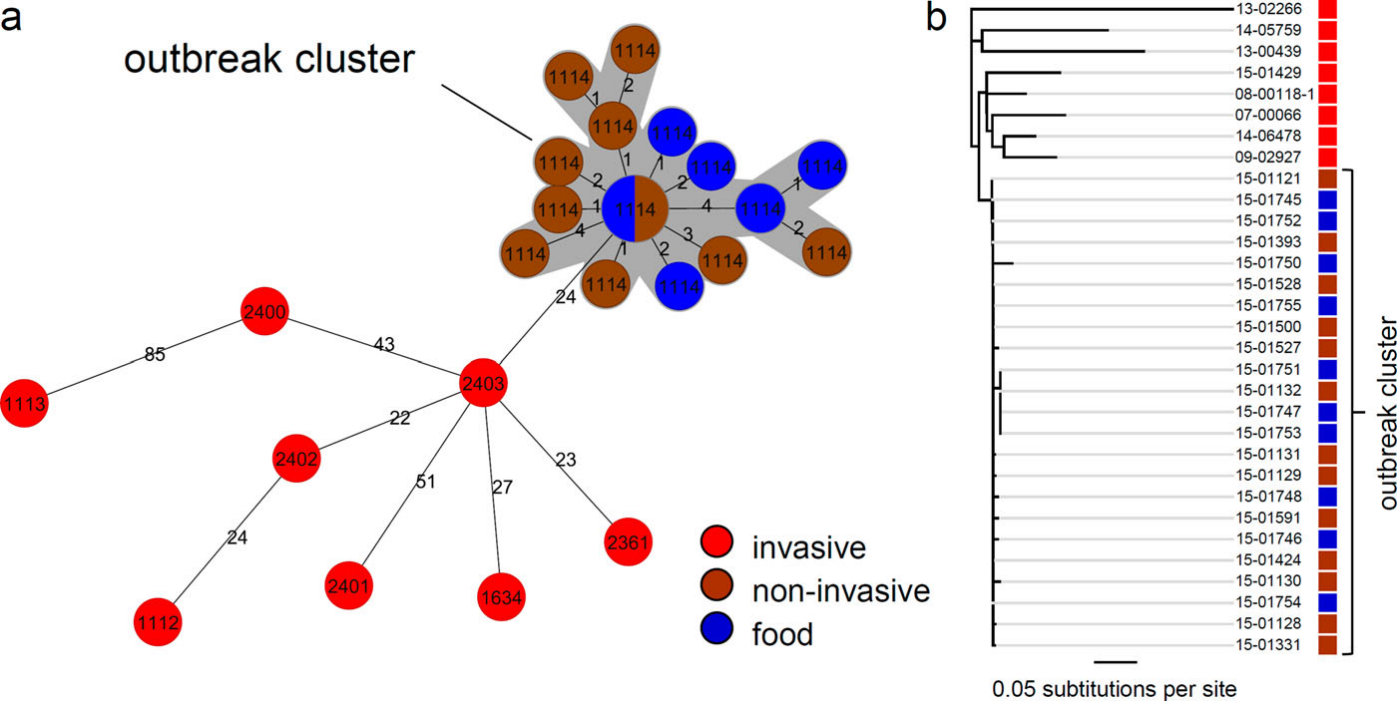
Confirmation of the outbreak cluster by genome sequencing. (a) Minimum spanning tree calculated using cgMLST data (based on Ruppitsch’s cgMLST scheme) of gastroenteritis outbreak isolates (brown), causative food isolates (blue) as well as selected isolates from epidemiologically unrelated invasive listeriosis cases (red). Please note that distances between nodes in minimal spanning trees cannot be summed up to calculate the number of different alleles of more distantly related isolates. Circle size corresponds to isolate number and CTs are indicated. Isolate pairs with allele differences ≤10 are considered to be epidemiologically linked [24]. (b) Neighbor-joining tree illustrating phylogenetic relatedness of the same set of isolates after mapping to the genome of the *L. monocytogenes* serotype 4b strain CLIP 80459 [28] as the reference and SNP filtering. Colour coding of isolates is the same as in panel A. Please see the online version of the article for a colored figure.

After implementation of WGS for improved cluster detection by the consiliary laboratory in 2015, genomes of selected 17b/25var isolates (14 human and 10 food isolates) were sequenced. Seven epidemiologically unlinked serogroup IVb isolates with similar but not identical PFGE profiles were included as outliers (Table 1, Figure S1). Six out of these seven outlier isolates came from known invasive listeriosis cases, in one case patient history was unknown. All isolates belonged to ST2. cgMLST analysis using the 1701 locus scheme of Ruppitsch et al. [27] grouped all gastroenteritis isolates and all food isolates in a single cluster with cluster type (CT) 1114 (Figure 1(a)). Allelic differences between the CT1114 food and gastroenteritis isolates were 0–9 (median 3). The eight isolates (four human, four food) that formed the central node of this cluster could even not be discriminated at all by cgMLST. Within the whole CT1114 cluster, the greatest genetic distance to this central node was ≤4 alleles and the greatest genetic distance between the two most remote isolates was 10 alleles. All outlier isolates fell outside this cluster with 24–89 alleles difference to isolate 15-01121, used as a reference isolate. Remarkably, isolate 15-01429, which showed a PFGE profile identical to the gastroenteritis isolates (Table 1, Figure S1) had a different CT (CT1634), differed from isolate 15-01121 in as many as 32 alleles and was thus classified as an epidemiologically unlinked clone.

Mapping of all sequence reads to the genome of the serotype 4b strain CLIP 80459 [28], which is the closest known CT1114 relative with a closed genome sequence, as the reference confirmed that the gastroenteritis isolates formed a joined cluster together with all food isolates with 0–13 single nucleotide polymorphisms (SNPs) difference (median 4), whereas all invasive isolates differed in 25–107 SNPs (median 40) from the gastroenteritis isolates (Figure 1(b)). This strongly indicates that the outbreak of non-invasive listeriosis among the preschool children was caused by consumption of the suspected food types.

### *In vitro* virulence of invasive and gastroenteritis outbreak isolates

**Figure 2.**
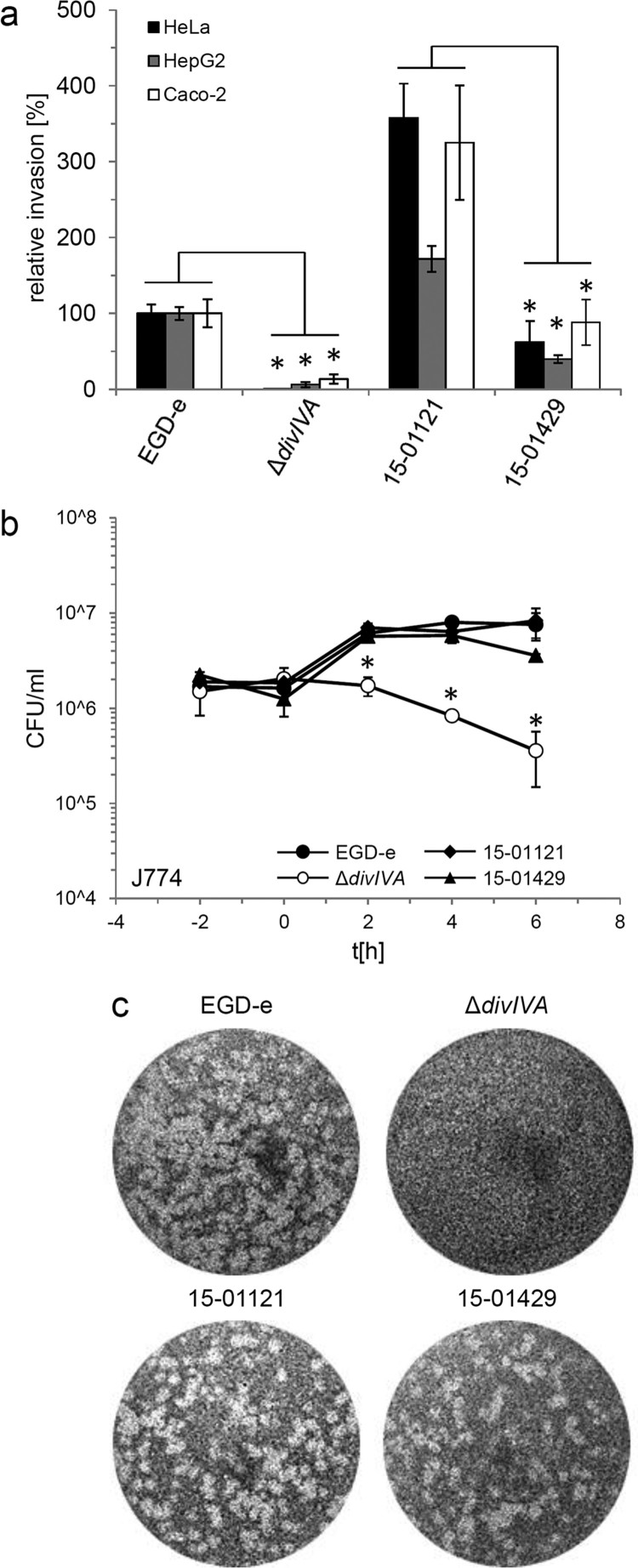
Invasion, intracellular multiplication and cell-to-cell spread of clinically invasive and gastroenteritis isolates. (a) Invasion assays. Isolates 15-01121 (gastroenteritis) and 15-01429 (invasive) were grown in BHI broth to mid-logarithmic growth phase and used to infect monolayers of HeLa, Hep-G2 and Caco-2 cells. Strains EGD-e and LMS2 (Δ*divIVA*) were included to control the experiment. The experiment was performed in triplicate, EGD-e values were set to 100%, average values are shown and standard deviations are indicated. Significant differences are indicated by asterisks (*t*-test, *P *< .01). (b) Intracellular multiplication of the same set of strains as in panel A in J774 mouse macrophages. Average values and standard deviations are shown, which were calculated from an experiment performed in triplicate. Significant differences are indicated by asterisks (*t*-test, *P *< .01). (c) Plaque formation assay to monitor the ability to spread from cell to cell in 3T3 mouse fibroblasts. The same set of strains as in panel A was tested.

In order to compare invasion rates of gastroenteritis and invasive isolates, isolates 15-01121 (gastroenteritis CT1114 reference isolate) and 15-01429 (invasive CT1634 isolate) were tested in *in vitro* invasion assays using the two human epithelial cell lines HeLa and Caco-2 and the human hepatocyte cell line Hep-G2. Unexpectedly, isolate 15-01121 was four to six times more invasive than isolate 15-01429 (Figure 2(a)). In contrast, an isogenic mutant of strain EGD-e lacking the *divIVA* gene, required for listerial cell division and invasion [29,30], was unable to invade any of these cell types (Figure 2(a)). Thus, the differences in disease outcome of the isolates tested here cannot be explained by differences in their potential to invade non-phagocytic human host cells.

Intracellular multiplication of the gastroenteritis (15-01121) and invasive isolate (15-01429) was tested in J774 A.1 murine macrophages. Both isolates multiplied within the macrophages to a similar degree as the EGD-e reference strain, but the number of colony forming units of the invasive isolate 15-01429 is reduced approximately two-fold at the last time point. In contrast, the Δ*divIVA* mutant was killed inside the macrophages (Figure 2(b)), as reported before [31]. Finally, no differences in cell-to-cell spread between the invasive and the gastroenteritis isolate were observed (Figure 2(c)). Taken together, disease outcome did not correlate with virulence phenotypes observed in *in vitro* infection assays.

### Genetic differences between invasive and gastroenteritis outbreak isolates

Sequence variations between invasive and gastroenteritis isolates were searched using a comparative genomics approach. To this end, genome sequences of isolates 15-01121 (gastroenteritis) and 15-01429 (invasive) were assembled from Illumina raw reads using Trimmomatic [32] and the A5-miseq pipeline [33], producing 17 or 13 contigs with a total number of 2.969.661 or 2.968.729 base pairs, respectively. Mapping of the 15-01121 genome sequence on 15-01429 as the reference genome and vice versa using Bowtie2 [34] identified 42 sequence variations within coding sequences that either caused amino acid exchanges, frameshifts or premature stop codons. This list of sequence variations was then filtered for those that are present in the genomes of all gastroenteritis and absent in the genomes of all invasive isolates tested here (or *vice versa*) and thus may be associated with disease outcome. Thirteen sequence polymorphisms remained and these caused 12 amino acid exchanges in 12 different genes and one stop codon in the *lmo0105* gene encoding the *L. monocytogenes* chitinase B (*chiB*) (Table S1).

Analysis of the virulome revealed the presence of LIPI-1 and the absence of LIPI-3 and LIPI-4 in the outbreak cluster strains (Figure S2). Furthermore, a similar pattern of internalin genes as in the serotype 4b strain F2365 [35] was observed. Importantly, no amino acid variations clustering with diseases outcome were detected in internalins and other genes contributing to invasion. However, a remarkable exception is a V957E exchange in *lmo0327* (Table S1), encoding an internalin-like autolysin [36,37] that is required for full expression of *chiA* and *chiB* in an unknown way [36].

### Gastroenteritis outbreak isolates do not produce ChiB

**Figure 3.**
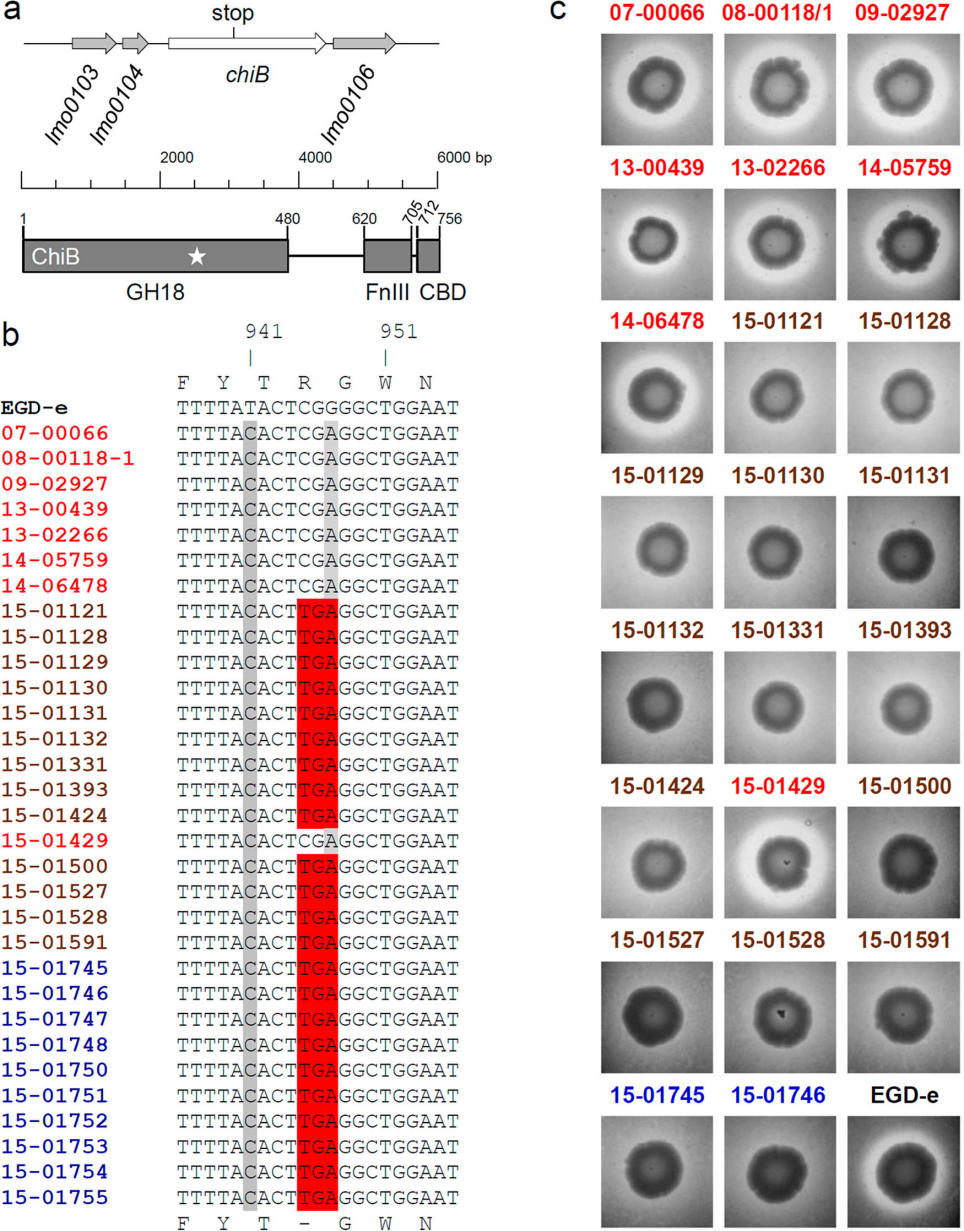
Chitinase activity of selected gastroenteritis outbreak isolates. (a) Schematic illustration showing the *lmo0105* (*chiB*) locus of *L. monocytogenes* EGD-e and the position of the premature stop codon in the gastroenteritis outbreak isolates (upper part). Domain organization of the ChiB protein according to Paspaliari et al*.* [60] (bottom part). The premature stop codon is indicated by a star. GH18 – family 18 glycoside hydrolases domain, FnIII – fibronectin type III-like domain, CBD – carbohydrate binding domain. (b) Multiple sequence alignment of the *chiB* gene sequences (partial) of all *L. monocytogenes* isolates sequenced in this study. The 315th codon of *chiB* (CGA, encoding arginine) is changed into the TGA stop codon in all gastroenteritis outbreak isolates and in all food isolates. (c) Chitinase activity of invasive isolates, gastroenteritis outbreak isolates and two selected food isolates on chitin agar plates after 4 days of incubation at 30°C. Halo formation indicates chitin hydrolysis. EGD-e was included as positive control. Please see the online version of the article for a colored figure.

The 315th *chiB* codon was changed from CGA (arginine) to TGA leading to premature translation termination (Figure 3(a)). This premature stop codon was present in all CT1114 gastroenteritis isolates as well as all CT1114 food isolates (Table S1) but was absent in all isolates, which were obtained from invasive listeriosis patients tested here (Figure 3(b)). The stop codon chopped off several essential protein domains including the chitin binding domain (Figure 3(a)). To test, whether this mutation caused impaired chitin hydrolysis, culture aliquots of all invasive and gastroenteritis isolates as well as two selected food isolates were spotted on BHI agar plates containing chitin. After four days of incubation all invasive isolates (containing the full-length *chiB* gene) produced halos indicating chitin hydrolysis, whereas all gastroenteritis isolates and the associated food isolates were unable to hydrolyse chitin (Figure 3(c)).

Chitinase production in the same set of isolates was further analysed by Western blotting. This showed that ChiB was only found in the supernatants of the invasive isolates and could not be detected in any of the isolates from the gastroenteritis outbreak. In contrast, ChiA was present in the supernatants of all tested isolates (Figure S3).

### Correction of chitinase production in a gastroenteritis outbreak isolate

**Figure 4.**
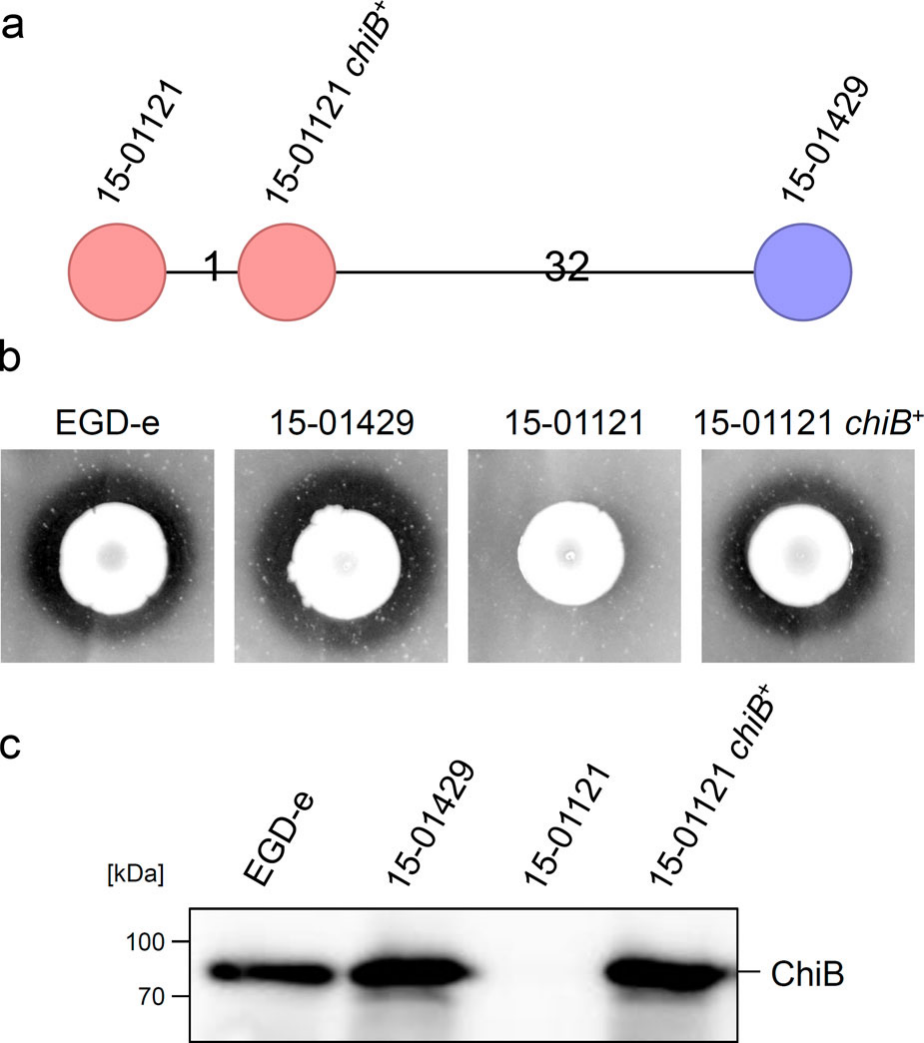
Restoration of ChiB production in the 15-01121 gastroenteritis isolate. (a) cgMLST analysis of the gastroenteritis isolate 15-01121, the invasive isolate 15-01429 and isolate 15-01121 after correction of *chiB*. (b) Chitinase production of strains 15-01429, 15-01121 and LMSB1 (15-01121 *chiB^+^*) on chitin containing BHI agar plates. (c) Western blot showing presence of ChiB in culture supernatants of the same set of strains as in panel A. Please see the online version of the article for a colored figure.

Next, the defective *chiB* allele of isolate 15-01121 containing the premature stop codon was exchanged against that of isolate 15-01429 to test whether ChiB affects virulence in our strain backgrounds. Genome sequencing confirmed exchange of the *chiB* allele in the resulting 15-01121-*chiB*^+^ strain (named LMSB1), which differed in one allele (i.e. in the corrected *chiB*) from its parental isolate 15-01121 after cgMLST (Figure 4(a)). As expected, strain 15-01121 *chiB^+^* showed chitinase activity on chitin containing agar plates (Figure 4(b)) and western blotting demonstrated that it secreted ChiB into the culture supernatant to a similar degree as the ChiB producing invasive isolate 15-01429 (Figure 4(c)). This shows that the *σ*^54^-dependent promoter (TGGCA-N_6_-TTGCA) [38] identified upstream of *chiB* in strain 15-01121 drives *chiB* expression under our culture conditions and indicates that the premature stop codon in the *chiB* gene of isolate 15-01121 was the sole reason for its inability to degrade chitin.

### Effect of *chiB* correction on virulence

**Figure 5.**
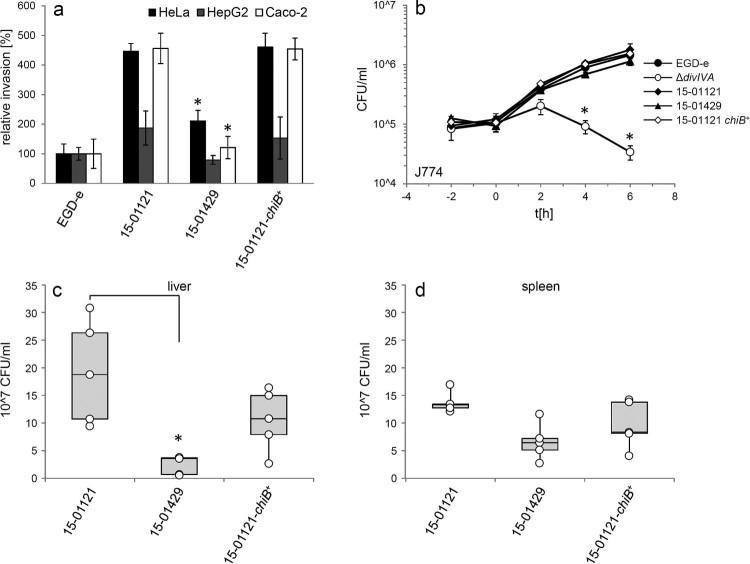
Effect of *chiB* correction on virulence. (a) Invasion of the gastroenteritis isolate 15-01121, the invasive isolate 15-01429 and the gastroenteritis isolate with the corrected *chiB* gene 15-01121-*chiB*^+^ into different cell types. *L. monocytogenes* strain EGD-e was included as control. EGD-e values were set to 100%, average values are shown and standard deviations are indicated. Significant differences compared to 15-01121 are indicated by asterisks (*t*-test, *P *< .01). (b) Intracellular growth of the same set of strains in J774 mouse macrophages. *L. monocytogenes* strains EGD-e and LMS2 (Δ*divIVA*) were included as controls here. All experiments were performed as triplicates and average values and standard deviations are shown. Significant differences are indicated by asterisks (*t*-test, *P *< .01). (c–d) Mouse infection experiment. C57BL/6 mice (*n* = 5) were infected via their tail vene with 5 × 10^4^ bacteria and the bacterial load in liver (c) and spleen (d) was determined on day 3 post infection. Data are represented as box plot and differences statistically significant according to the Brown–Forsythe test are indicated with asterisks.

Infection experiments using human Hep-G2 hepatocytes did not reveal differences in intracellular multiplication between the 15-01121, 15-01429 and the 15-01121-*chiB^+^* strains (Figure S4(a)). Likewise, correction of chitinase production did neither alter adhesion to HeLa cells (Figure S4(b)) nor did it alter invasion of strain 15-01121-*chiB*^+^ into HeLa, Hep-2G or Caco-2 cells when compared to isolate 15-01121 (Figure 5(a)). Moreover, no differences in intracellular growth were observed with strain 15-01121-*chiB*^+^ in infection experiments using J774 or RAW264.7 mouse macrophages, whereas the Δ*divIVA* mutant, used as a negative control here, could clearly not multiply in these cells (Figure 5(b), Figure S4(c)). These results were in good agreement with the work of Chaudhuri et al*.* [20], who reported that *chiB* had no effect on virulence in *in vitro* assays when deleted in the *L. monocytogenes* reference strain 10403S (serotype 1/2a, lineage II). However, the same *L. monocyctogenes* 10403S *chiB* mutant proliferated 8–14-fold less within the liver and spleens of mice compared to wild type [20]. We thus tested bacterial replication of strains 15-01121, 15-01121-*chiB*^+^ and 15-01429 in C57BL/6 mice. For this purpose, five animals per strain were infected with 5 × 10^4^ bacterial cells via their tail veins and sacrificed at the third day post infection. In contrast to our expectation, the ChiB-deficient gastroenteritis isolate 15-01121 had multiplied 7.8-fold and two-fold more in liver and spleen, respectively, when compared to the invasive ChiB-secreting isolate 15-01429. Most remarkably, ChiB production in strain 15-01121-*chiB*^+^ did not significantly affect bacterial proliferation within the animal organs, when compared to its parental isolate 15-01121 (Figure 5(c–d)). These data indicate that chitinase ChiB does not affect virulence towards C57BL/6 mice in the background of isolate 15-01121 under the conditions tested here.

## Discussion

Even though listeriosis is primarily considered an invasive disease only affecting members of susceptible risk groups, non-invasive gastroenteritis due to *L. monocytogenes* infection is not an uncommon manifestation in healthy individuals. Such gastroenteritis cases are often accompanied by fever and typical gastrointestinal symptoms such as non-bloody diarrhoea, vomiting or nausea and are characterized by a rapid onset of disease within less than 24 h from the time of ingestion [9]. Sporadic cases of *L. monocytogenes* gastroenteritis seem to be rather rare events [39], but several gastroenteritis outbreaks due to *L. monocytogenes* infection had been reported. These outbreaks are typically caused by consumption of food products contaminated with high bacterial loads of *L. monocytogenes* serotype 1/2a [12,40,41], 1/2b [11,42,43] or 4b strains [10,44]. The largest reported *L. monocyctogenes* gastroenteritis outbreak occurred in Italy with more than 1500 affected and almost 300 hospitalized patients during spring 1997 [10]. Listerial gastroenteritis is usually a self-limiting disease and progression into invasive listeriosis is only reported in a small number of patients [9,11].

Most of our understanding of listerial disease mechanisms concerns its ability to cause invasive disease. In contrast, little is known regarding the virulence mechanisms used by *L. monocytogenes* during non-invasive disease [9]. Only recently, the surface protein ActA, required for actin tail formation during intracellular passages has been shown to contribute to gut colonization in mice by mediating bacterial auto-aggregation [45]. Furthermore, the bile salt hydrolase Bsh mediates hydrolysis of antibacterial bile salts and supports persistence of *L. monocytogenes* in the gastrointestinal tract [46]. However, it seems unlikely that the presence or absence of a special set of virulence genes is responsible for the non-invasive disease manifestations in the gastroenteritis cluster for several reasons: First, all gastroenteritis patients affected by the outbreak here were either of preschool age or healthy adults and thus did not belong to the risk group susceptible for invasive disease, further emphasizing the important role of the host immune status for progression of non-invasive into invasive listeriosis. Second, assuming a serving size of 100 g, the bacterial load in the contaminated rice pudding (1.5 × 10^9^ CFU) was at the upper end of the estimated infectious dose range for the non-risk population (10^7^–10^9^ CFU) and thus sufficient to induce infection even in healthy individuals. Third, no sequence variations were found in the classical virulence factors. Fourth, a representative isolate from the gastroenteritis disease cluster was even more successful in mice and cell culture infections when compared to a closely related isolate obtained from an invasive listeriosis patient and virulence of the gastroenteritis isolate did not change after introduction of the active *chiB* allele. While it would be interesting to know whether differences in virulence might be detected between the gastroenteritis and invasive isolates in animal models for *L. monocytogenes* gastroenteritis, our results already demonstrate that the chitinase ChiB is dispensable for *L. monocytogenes* to cause gastroenteritis in humans. It remains, however, unclear whether the lack of chitinase activity had a protective role in prevention of further disease progression.

The two listerial chitinases, ChiA and ChiB, are synergistically required for chitinase activity [21] and their expression is induced during growth in the soil [22], but their role for virulence is not entirely clear. ChiA and ChiB have contributed to replication of the reference strain 10403S in mice [20]. In a later report, Δ*chiA* mutants in the same background were cleared more efficiently from infected mice, presumably due to unrepressed expression of iNOS, the inducible NO synthase [23]. As mammalian hosts do not synthesize chitin, carbohydrate chains in glycoproteins, proteoglycans or glycolipids with structural similarity to chitin could be the substrates of bacterial chitinases [47,48]. Human chitinases possess a variety of immune-modulating functions [49] and thus, listerial chitinases may modulate the activity of a glycosylated factor that controls iNOS expression [23]. That we do not observe a similar effect in our strains, indicates that these findings cannot simply be generalized to other genetic strain backgrounds and animal models.

We analysed *chiB* sequences of 2211 *L. monocytogenes* genomes available at NCBI and ∼1200 genome sequences of *L. monocytogenes* isolates from human infections in Germany currently present in our database. The *chiB* gene occurred in 206 different alleles including four alleles with premature stop codons, all of which lie in the catalytic GH18 domain (Figure 3(a)), and terminate ChiB translation after the 100th, 314th (as in the gastroenteritis strains), 344th or 452nd codon. These mutations occurred in seven NCBI genomes with unknown origin, a single sporadic German serogroup ST2 isolate (strain ID: 17-02631, CT5652) from 2017 and a set of 11 German ST4 strains isolated in 2017–2018 (Figure S5(a)). The German ST2 isolate is a close relative of the outbreak reference isolate 15-01121 (15 different alleles according to cgMLST). It had the same truncation in its *chiB* allele as the gastroenteritis isolates and lacked detectable chitinase activity (Figure S5(b)), but was isolated from blood of an 80 years old patient. Likewise, 10 out of the 11 ST4 strains with truncated *chiB* alleles were isolated from blood or cerebrospinal fluid of listeriosis patients (the source of isolation of the 11th isolate is not known) and also lacked detectable chitinase activity (Figure S5(b)). Even though these are rare events, this suggests that chitinase ChiB can even dispensable for invasive listeriosis in humans.

## Materials and methods

### Bacterial strains and growth conditions

All *L. monocytogenes* isolates are listed in Table 1. Strains EGD-e [50] and its isogenic mutant lacking the *divIVA* gene [29] were used as controls. *L. monocytogenes* strains were routinely cultivated in BBL^TM^ brain heart infusion (BHI) broth (Becton Dickinson) or on BHI agar plates at 37°C if not stated otherwise. Bacterial counts in food matrices were assessed according to DIN EN ISO 11290-2 [51].

### Determination of molecular serogroups and PFGE

Molecular serogroups were determined by multiplex PCR [52,53]. PFGE was performed using the PulseNet protocol (https://www.cdc.gov/pulsenet/pdf/listeria-pfge-protocol-508c.pdf). AscI and ApaI restriction patterns were analysed with BioNumerics software, version7.1 (Applied Maths BVBA, Sint-Martens-Latem, Belgium).

### Genome sequencing, MLST and core genome MLST

Standard methods were used for isolation of chromosomal DNA [54]. One nanogram of genomic DNA was used for library generation by the Nextera XT DNA Library Prep (Illumina). Sequencing was carried out on a MiSeq benchtop sequencer and performed in paired-end modus (2 × 300 bp) using a MiSeq Reagent Kit v3 cartridge (600-cycle kit). Reads were mapped against the 1701 targets of the *L. monocytogenes* core genome MLST scheme [27], using the Ridom SeqSphere Software (Münster, Germany). Sequence types (STs) and CTs were determined after automated allele submission to the cgMLST server for *L. monocytogenes* (http://www.cgmlst.org/ncs/schema/690488/). Minimum spanning trees were calculated in the ‘pairwise ignore missing values’ mode.

### Virulome analysis

Based on the virulome gene set of Moura et al. [55], 98 genes previously shown to be important during infection were included as target loci in a SeqSphere task template. Reference sequences for LIPI-3 were taken from the F2365 genome (NC_002973.6), for LIPI-4 from the CLIP 80459 genome (NC_012488.1) and all other from the genome of EGD-e (NC_003210). Raw reads were mapped against them using SeqSphere and alleles were considered as present when identity was >90% and at least 99% of the reference sequence aligned with the query sequence.

### SNP-based alignment

Mapping of sequencing reads, generation of consensus sequences and alignment calculation was performed using the BatchMap in-house pipeline [24]. *L. monocytogenes* CLIP 80459 [28] was used as the reference sequence and SNPs were filtered using an exclusion distance of 300. Nucleotide positions that were invariable, containing gaps or ambiguities were stripped from the alignment before distance calculation and clustering was performed using the Geneious 9.1.3 Tree builder (Biomatters Ltd).

### Genome comparisons

For identification of differences between genomes, sequencing reads were trimmed using Trimmomatic [32] with default parameters and mapped against the assembled genomes of the sequenced isolates using Bowtie2 with −X 2000 [34]. Assembly of the sequenced reads was done using the A5-miseq assembler pipeline [33]. Annotation of coding sequences was transferred automatically from the EGD-e genome (NC_003210) using Geneious 9.1.3 (Biomatters Ltd). SNPs were detected and filtered in Geneious. All called SNPs were manually inspected to discard false positives, elucidate SNP locations and the potential impact on predicted coding sequences.

### Plasmid and strain construction

The *chiB* gene of isolate 15-01429 was amplified using the primers SB3 (GCGCGCGAATTCATGAAAAAGCTTTTTAGTATTACTTCTG) and SB4 (GCGCGCAGATCTTTATTAACAACCAAGGACCCCAC) and cloned with EcoRI/BglII into the EcoRI/BamHI cut pMAD backbone of plasmid pSH246 [56]. The insert DNA sequence of the resulting plasmid (pSB2) was verified by Sanger sequencing. Plasmid pSB2 was used to transform isolate 15-01121 by electroporation [57] and the defective endogenous chromosomal *chiB* allele of isolate 15-01121 was then exchanged against the wild type *chiB* allele of pSB2 by homologous recombination. Correction of the *chiB* stop codon in the resulting strain (LMSB1) was verified by genome sequencing that was performed as described above.

### Chitinase assay

Chitinase activity was determined using a previously published protocol with minor modifications [58]. For the production of chitinase BHI agar plates, 5 g chitin from shrimp shells (Sigma-Aldrich) were dissolved in 50 ml 37% HCl. The colloidal chitin suspension was neutralized using concentrated NaOH and washed five times with sterile PBS. One hundred and eighty millilitre BHI agar were mixed with 20 ml colloidal chitin suspension (10 g/l chitin final concentration). *L. monocytogenes* strains were grown in BHI broth at 37°C and spotted onto chitin agar plates. Plates were incubated at 30°C until halo formation becomes visible (usually three to six days).

### Detection of listerial chitinases

*L. monocytogenes* strains were grown in LB broth to OD_600_ = 1.0. Extracellular proteins were isolated as described [59], separated by standard SDS polyacrylamide electrophoresis and transferred onto positively charged polyvinylidene fluoride membranes. Immune-staining was carried out using rabbit polyclonal antisera against full-length ChiA and ChiB [58] as the primary, and anti-rabbit immunoglobuline G conjugated to horseradish peroxidase as the secondary antibody. The peroxidase conjugates were visualized on the membrane by the ECL chemiluminescence detection system (Thermo Scientific, Rockford, IL, USA).

### *In vitro* virulence assays

Invasion of *L. monocytogenes* strains into HeLa, Caco-2 and Hep-G2 cells was determined as described in detail elsewhere [29]. Shortly, 10^5^ cells were seeded into the wells of a 24 multi well plate and infected with 2 × 10^6^ bacteria. The bacteria were allowed to invade the cells and extracellular bacteria were first washed off and the remaining extracellular bacteria were killed by gentamicin treatment. Sampling was performed by lysing the infected cells in 1 ml of ice-cold PBS containing 0.1% Triton X-100. In order to measure the number of recovered bacteria, serial dilutions were plated on BHI agar plates incubated over night at 37°C. Assays for determination of adhesion rates were performed in the same way, but without the gentamicin step used to kill extracellular bacteria. Intracellular growth inside J774 mouse macrophages and cell-to-cell spread using 3T3 mouse embryo fibroblasts in the plaque formation assay were essentially carried out as described earlier [29,59]. Infection assays using RAW264.7 macrophages were performed in the same way as those using J774 macrophages.

### Animal experiments

Age and sex matched C57BL/6 WT mice were obtained from Janvier (Le Genest Saint Isle, France). All animals were kept under conventional conditions in an isolation facility throughout the experiments. *L. monocytogenes* strains were grown in BHI broth and aliquots of log-phase cultures were stored at −80°C. For i.v. infection, fresh log-phase cultures were prepared from frozen stocks and 5 × 10^4^*L. monocytogenes* diluted in 200 µl sterile pyrogen-free PBS (pH 7.4) were injected. The bacterial dose used for infection was controlled by plating an inoculum on BHI agar and counting colonies after incubation at 37°C for 24 h. To determine CFUs in *L. monocytogenes* infected mice, organs were dissected and homogenized with sterile tissue grinders on day 3 p.i. Ten-fold serial dilutions of the homogenates were plated on BHI agar. Bacterial colonies were counted microscopically after incubation at 37°C for 24 and 48 h. All animal experiments were in compliance with the German Animal Welfare Act (TierSchG) in a protocol approved by the Landesverwaltungsamt Sachsen-Anhalt (file number: 203.h-42502-2-901, University of Magdeburg).

### Data availability

Genome sequencing raw files are available at the European nucleotide archive (https://www.ebi.ac.uk/ena) under study accession numbers PRJEB24496 [24] and PRJEB26654.
